# Mechanisms of GOLPH3 Associated with the Progression of Gastric Cancer: A Preliminary Study

**DOI:** 10.1371/journal.pone.0107362

**Published:** 2014-10-06

**Authors:** Jinzhen Peng, Ye Fang, Yong Tao, Keke Li, Ting Su, Yuncui Nong, Fang Xie, Mingyu Lai

**Affiliations:** 1 Department of Geriatric Gastroenterology, the First Affiliated Hospital of Guangxi Medical University, Nanning, Guangxi, P. R. China; 2 Department of Spine Osteopathia, the First Affiliated Hospital of Guangxi Medical University, Nanning, Guangxi, P. R. China; Taipei Medical University, Taiwan

## Abstract

**Study Design:**

To investigate the specific mechanisms by which Golgi phosphoprotein 3 (GOLPH3) affects the progression of gastric cancer and to explore its clinical significance.

**Methods:**

Immunohistochemical analysis was used to evaluate the correlations between GOLPH3, phosphorylated mTOR (p-mTOR), phosphorylated Akt (p-Akt), phosphorylated p70S6 (p-p70S6), phosphorylated 4E-BP1 (p-4E-BP1) and the clinicopathological features of gastric cancer. The mRNA expression levels of GOLPH3, mTOR, Akt, p70S6 and 4E-BP1 in gastric cancer, carcinoma-adjacent and paired normal tissue were analyzed using RT-PCR. Western blotting was used to determine the protein expression of GOLPH3, p-mTOR, p-Akt, p-p70S6 and p-4E-BP1 in tissues.

**Results:**

High expression protein levels of GOLPH3, p-AKT, p-mTOR, p70S6, p-4E-BP1 were positively associated with histological grade (*p*<0.05), depth of invasion (*p*<0.05), distant metastasis (*p*<0.05) and lymph node involvement (*p*<0.05). Compared with carcinoma-adjacent and paired normal tissues, the mRNA expression levels of GOLPH3, AKT, mTOR, p70S6 and 4EBP1 in gastric cancer tissues were significantly higher. The protein expression levels of GOLPH3, p-AKT, p-mTOR, p-p70S6 and p-4E-BP1 in gastric cancer tissues were also significantly higher than in carcinoma-adjacent and paired normal tissues. A strong positive correlation was observed between GOLPH3, p-mTOR, p-p70S6 and p-4EBP1 expression (*r* = 0.410, 0.303 and 0.276, respectively, *p*<0.05), but no significant correlation between the expression of GOLPH3 and p-Akt was observed.

**Conclusions:**

The GOPLH3 expression level is highly correlated with Akt/mTOR signaling in human gastric cancer samples. GOLPH3 combined with Akt/mTOR signaling activation may play an important role in the development, differentiation, invasion and metastasis of gastric cancer.

## Introduction

Although global statistics show that gastric cancer (GC) is the fourth most common cancer in men and the fifth most common cancer in women, the mortality from gastric cancer ranks second only to lung cancer [1]. The global incidence of gastric cancer has been declining since World War II [2]. However, in developing nations, including China, the morbidity and mortality of gastric cancer have remained high. Gastric cancer treatment has improved in recent years, but most patients are still diagnosed with advanced gastric cancer, frequently including invasion and distant metastasis, leading to an unsatisfactory treatment effect rate. Because gastric cancer presents a significant threat to human health and life, it is crucial to understand the pathogenesis of gastric cancer in the process of tumorigenesis, invasion and metastasis to guide early diagnosis and treatment.

It is commonly accepted that tumor formation involves the misregulation of numerous oncogenes, tumor suppressor genes and metastasis-related genes Golgi phosphoprotein 3 (GOLPH3), which is also known as GPP34, GMx33, or MIDAS, is a 34-kD membrane protein that was initially identified in the mouse Golgi apparatus by proteomic analysis [3]. GOLPH3 belongs to the Golgi matrix protein family, which is highly conserved from yeast to humans [4]. After GOLPH3 has been translationally modified, it is dynamically connected to the negative Golgi matrix, rapidly transported through the trans-Golgi network (TGN) to the cytoplasm, and distributed into plasma membrane vesicles and endocrine cells [4]. Several studies have demonstrated that GOLPH3 is involved in anterograde and retrograde Golgi trafficking, receptor recycling, and glycosylation from the Golgi apparatus to the plasma membrane; GOLPH3 also plays a role in cytoskeletal interactions and maintenance of the Golgi structure [4]–[7]. Scott et al. first identified the oncogenic role of GOLPH3 by revealing its important role in tumor cell differentiation and proliferation and its presence in many solid cancers [8]. Interestingly, some studies have suggested that GOLPH3 regulates cancer cells by enhancing mammalian target of rapamycin (mTOR) activity. mTOR is a serine/threonine protein kinase and a key integrator of phosphatidyl-inositol-3 kinase (RTK-PI3K) pathways known to regulate cell growth, proliferation, and survival in human cancer cells [9]–[10]. Many studies are currently investigating the relationship between the GOLPH3 and several solid cancers However, there is insufficient evidence to support the hypothesis that GOLPH3 expression is associated with human gastric cancer progression and prognosis, and the mechanistic basis by which GOLPH3 affects the tumorigenesis, invasion and metastasis of gastric cancer remains unclear. In this study, we investigated clinical significances of GOLPH3 and AKT/mTOR signaling in gastric cancer. Meanwhile, we discovered the relation of GOPLPH3 and Akt/mTOR signaling in gastric cancer to reveal the potential role of GOLPH3 in regulating mTOR-mediated gastric cancer tumorigenesis, invasion and metastasis.

## Materials and Methods

### Ethical statement

All study procedures were reviewed and approved by the Institutional Ethics Review Board of the First Affiliated Hospital of Guangxi Medical University and conducted according to the principles expressed in the Declaration of Helsinki. Informed consent was exempted by the board due to the fresh specimens were handled and anonymized according to ethical and legal standards.

### Tissue samples and clinical data selection

The fresh tissue samples were collected from eighty patients with gastric cancer who underwent surgical treatment at the First Affiliated Hospital of Guangxi Medical University during the period from January 2012 to January 2013. Cancerous and carcinoma-adjacent tissues (3-cm distance from the cancer) and paired normal tissues were obtained from each patient, and the patient was diagnosed independently by two experienced pathologists according to the American Joint Committee on Cancer criteria [11]. Complete original clinical data were available for patients diagnosed with gastric cancer. The group included 36 females and 44 males, with an average age of 55.4±13.1 years (range 27–75 years). Additional patient characteristics are shown in Table 1, Table 2 and Table 3. Patients receiving chemotherapy or radiotherapy prior to surgery or who had a history of other tumors were excluded. In the following studies, a portion of the specimen taken during surgery was immediately snap-frozen in liquid nitrogen and subsequently stored at −80°C, and a portion was fixed at 10% buffered formalin for 24 h and embedded in paraffin.

**Table 1:** Correlation between clinicopathological features and the GOPLH3, p-mTOR expression in gastric cancer specimen.

Clinicopathologicalvariable	Number	GOLPH3 expression			p-mTOR expression		
		High	Low	p value	High	Low	p value
**Age (years)**				0.801			0.700
≥55	46	27	19		25	21	
<55	34	19	15		17	17	
**Gender**				0.833			0.917
Male	51	34	17		27	24	
Female	29	20	9		15	14	
**Histological grade**				0.011*			0.030*
Well/moderate	36	20	16		15	21	
Poorly	44	36	8		29	15	
**Depth of invasion**				0.007*			0.001*
T1/T2	39	17	22		23	26	
T3/T4	41	30	11		33	8	
**Lymph node metastasis**				0.004*			0.015*
NO	38	8	30		16	22	
YES	42	31	11		29	13	
**Distant metastasis**				0.002*			0.022*
NO	53	20	33		23	30	
YES	27	20	7		19	8	

The associations between protein expression and clinicopathological variables were estimated using the chi-squared test.

*Significant at the level of *p*<0.05.

**Table 2:** Correlation between clinicopathological features and the p-AKT1, p-4E-BP1 expression in gastric cancer specimen.

Clinicopathologicalvariable	Number	p-AKT1 expression			p-4E-BP1 expression		
		High	Low	p value	High	Low	p value
**Age (years)**				0.094			0.518
≥55	46	30	16		29	17	
<55	34	21	23		19	15	
**Gender**				0.368			0.054
Male	51	30	21		22	29	
Female	29	20	9		19	10	
**Histological grade**				0.001*			0.027*
Well/moderate	36	13	23		14	22	
Poorly	44	32	12		28	16	
**Depth of invasion**				0.001*			0.000*
T1/T2	39	18	21		13	26	
T3/T4	41	34	7		30	11	
**Lymph node metastasis**				0.000*			0.000*
NO	38	10	28		15	23	
YES	42	32	10		33	9	
**Distant metastasis**				0.015*			0.009*
NO	53	22	31		25	28	
YES	27	19	8		21	6	

The associations between protein expression and clinicopathological variables were estimated using the chi-squared test.

*Significant at the level of *p*<0.05.

**Table 3:** Correlation between clinicopathological features and the p-P70S6 expression in gastric cancer specimen.

Clinicopathologicalvariable	Number	p-P70S6 expression		
		High	Low	p value
**Age (years)**				0.702
≥55	46	29	17	
<55	34	20	14	
**Gender**				0.555
Male	51	30	21	
Female	29	19	10	
**Histological grade**				0.005*
Well/moderate	36	17	19	
Poorly	44	34	10	
**Depth of invasion**				0.002*
T1/T2	39	14	25	
T3/T4	41	29	12	
**Lymph node metastasis**				0.000*
NO	38	11	27	
YES	42	33	9	
**Distant metastasis**				0.015*
NO	53	22	31	
YES	7	19	8	

The associations between protein expression and clinicopathological variables were estimated using the chi-squared test.

*Significant at the level of *p*<0.05.

### Immunohistochemistry

Paraffin-embedded specimens were sectioned into 3-µm-thick sections from the cancerous, carcinoma-adjacent, and paired normal tissue samples and mounted on glass slides. The sections were initially baked at 65°C for 2 h. Then, the sections were deparaffinized in 100% xylene and rehydrated in a descending ethanol series (100%, 90%, 80%, and 70% ethanol) and distilled water according to standard protocols. Thereafter, the sections were soaked in the citrate antigenic retrieval buffer, heated in an autoclave, and allowed to cool to room temperature. After the sections were washed in PBS three times for five minutes, 3% hydrogen peroxide was added to block endogenous peroxidase activity and non-specific antigen binding at room temperature for 20 min. After washing again with PBS, the sections were incubated with a specific antibody in a moist chamber overnight at 4°C. The following primary antibodies were used: GOLPH3 antibody at 1∶100, p-mTOR antibody at 1∶200, p-Akt antibody at 1∶200, p-p70S6 antibody at 1∶100, and p-4E-BP1 antibody at 1∶200. For the negative control, the primary antibody was replaced with PBS, and the remaining steps were performed as previously described. The samples were then washed with PBS, and a polymer-reinforcing agent was added to the sections at room temperature for 20 min. After washing, the tissue sections were treated with HRP-tagged goat anti-rabbit IgG, biotinylated anti-rabbit immunoglobulin, and streptavidin peroxidase complex reagent for 30 min. The sections were then visualized in fresh 3-3′ diaminobenzidine solution, hematoxylin counterstain, and 0.1% hydrochloric acid (to aid color separation). Finally, the slides were mounted in neutral gum and analyzed with a bright-field microscope.

The degree of immunostaining of each tissue section was assessed independently by two experienced pathologists who were blind to the patients’ clinical data. The protein expression levels were evaluated using a semi-quantitative scoring system based on the total percentage of positively stained tumor cells and the staining intensity. The percentage of positively stained cells was scored according to the following criteria: 0 (≤5% positive tumor cells stained), 1 (6–25% positive tumor cells stained), 2 (26–50% positive tumor cells stained), 3 (≥51% positive tumor cells stained). The staining intensity was scored using a scale from 0 to 3 as follows: 0 (no staining), 1 (weak staining = light yellow), 2 (moderate staining = yellow-brown), 3 (strong staining = brown). For the analysis, aggregate scores <4 were defined as low expression, and scores ≥4 were defined as high expression.

### Reverse transcription polymerase chain reaction

Total RNA was extracted from fresh tissues using TRIzol reagent (Invitrogen, Carlsbad, CA, USA) according to the manufacturer’s instructions. The RT-PCR primers used in the study were designed and synthesized by Shanghai Sangon Biological Engineering Technology & Services Co. Ltd. β-Actin was used as an internal reference. Information regarding the primer sequences used in the study is shown in Table 4. The RT-PCR was performed using a two-step method. One microgram of total RNA was reverse-transcribed into cDNA at 37°C for 60 min and 85°C for 5 s. PCR was then performed according to the following conditions: heating to 95°C for 5 min; 40 cycles of denaturation for 30 s at 95°C, annealing at 60°C for 30 s, and extension for 30 s at 72°C; and final extension at 72°C for 7 min before the reaction was stored at 4°C. Seven micrograms of the final PCR product were loaded on a 2% agarose gel for electrophoresis and imaging analysis under ultraviolet light. The Quantity One software program (Bio-Rad, Hercules, CA, USA) was used to measure the bands and the β-actin gray value of the PCR products.

**Table 4:** Sequences of primers used for RT-PCR.

Molecule	Sequences (5′-3′)	Length
GOLPH3	Sense: 5′-TGTAAGTCAGATGCTCCAACAGG-3′	316 bp
[GenBank:64083]	Antisense: 5′-TCACCCATTTGTCAAGAACGG-3′	
AKT1	Sense: 5′-CAACTTCTCTGTGGCGCAGTG-3′	561 bp
[GenBank:207]	Antisense: 5′-GACAGGTGGAAGAACAGCTCG-3′	
mTOR	Sense: 5′-GGATGGCAACTACAGAATCAC-3′	309 bp
[GenBank:2475]	Antisense: 5′-TTATTTAGGGCCTCTGGTTTCA-3′	
4E-BP1	Sense: 5′-ACCGGAAATTCCTGATGGAG-3′	156 bp
[GenBank:1978]	Antisense: 5′-CCCGCTTATCTTCTGGGCTA-3′	
p70S6	Sense: 5′-TACTTCGGGTACTTGGTAA-3′	188 bp
[GenBank:6198]	Antisense: 5′-GATGAA GGGATGCTTTACT-3′	
β-actin	Sense: 5′-CTGGGACGACATGGAGAAAA-3′	564 bp
[GenBank:60]	Antisense: 5′-AAGGAAGGCTGGAAGAGTGC-3′	
β-actin	Sense: 5′-CCTAGAAGCATTTGCGGTGG-3	416 bp
[GenBank:60]	Antisense: 5′-AGCTACGAGCTGCCTGACG-3	

### Western blotting

The cancerous, carcinoma-adjacent and paired normal tissue samples were weighed and ground into small pieces in liquid nitrogen. Pre-chilled PBS was used to wash the specimens, which were then centrifuged at 5000 rpm at 4°C for 5 min. After washing three times with PBS, pre-chilled phosphate lysis buffer and PMSF were added. The sample was sonicated at 180 W at 4°C for 5 min and centrifuged at 1,000 rpm at 4°C for 5 min; the supernatant was then immediately removed, and the protein concentration was quantified by the Bradford assay using a commercial kit purchased from Bio-Rad Laboratories. The supernatants were mixed with loading buffer and boiled at 100°C for 5 min to denature the proteins. Equal quantities of each sample were loaded into the wells of a 4–12% sodium dodecylsulfate-polyacrylamide gel, electrophoresed, and transferred to a polyvinylidene fluoride membrane. The membrane was blocked with 5% BSA buffer for 1 h with shaking and then incubated at 4°C overnight with the individual primary antibodies diluted in TBST. The following primary antibodies were used: polyclonal rabbit anti-human GOLPH3 (1∶1000, Abcam plc, Cambridge, UK), polyclonal rabbit anti-human p-p70S6 (Thr389) (1∶1000, Cell Signaling Technology, Inc. Beverly, MA, USA), polyclonal rabbit anti-human p-Akt (Ser473) (1∶1000, Cell Signaling Technology, Inc. Beverly, MA, USA), monoclonal rabbit anti-human p-mTOR (Ser2448) (1∶1000, Cell Signaling Technology, Inc. Beverly, MA, USA) and monoclonal rabbit anti-human p-Akt (1∶1000, Cell Signaling Technology, Inc. Beverly, MA, USA). The membrane was then washed with TBST five times for five min each, and horseradish peroxidase-conjugated goat anti-rabbit IgG secondary antibody (Santa Cruz Biotechnology, SC-2004) was added prior to incubation at room temperature for 1.5 h with shaking. β-actin was used as an internal control. After washing with TBST three times for five min, the band of interest was detected using the ECL prime Western blotting kit (Shanghai Pufei Biotechnology, Shanghai, China), and the Quantity One analysis software (Bio-Rad, CA, USA) was used to evaluate the densitometry data.

### Statistical analyses

All of the statistical analyses were performed using the Statistical Package for the Social Sciences, version 17.0 (SPSS Inc., Chicago, IL, USA). The RT-PCR and Western blotting results are presented as the mean ± SE. A one-way ANOVA followed by LSD multiple-range tests was used to analyze the differences between groups. The relationships between GOLPH3 and p-mTOR, p-Akt, p-4E-BP, and p-p70S6 expression levels were estimated using Pearson’s correlation coefficient. The associations between protein expression and clinicopathological variables were analyzed using the chi-squared test. P<0.05 (two-tailed) was used as the level of statistical significance.

## Results

### Relationships between the clinicopathological variables and the protein expression levels by immunohistochemistry

**Figure 1:**
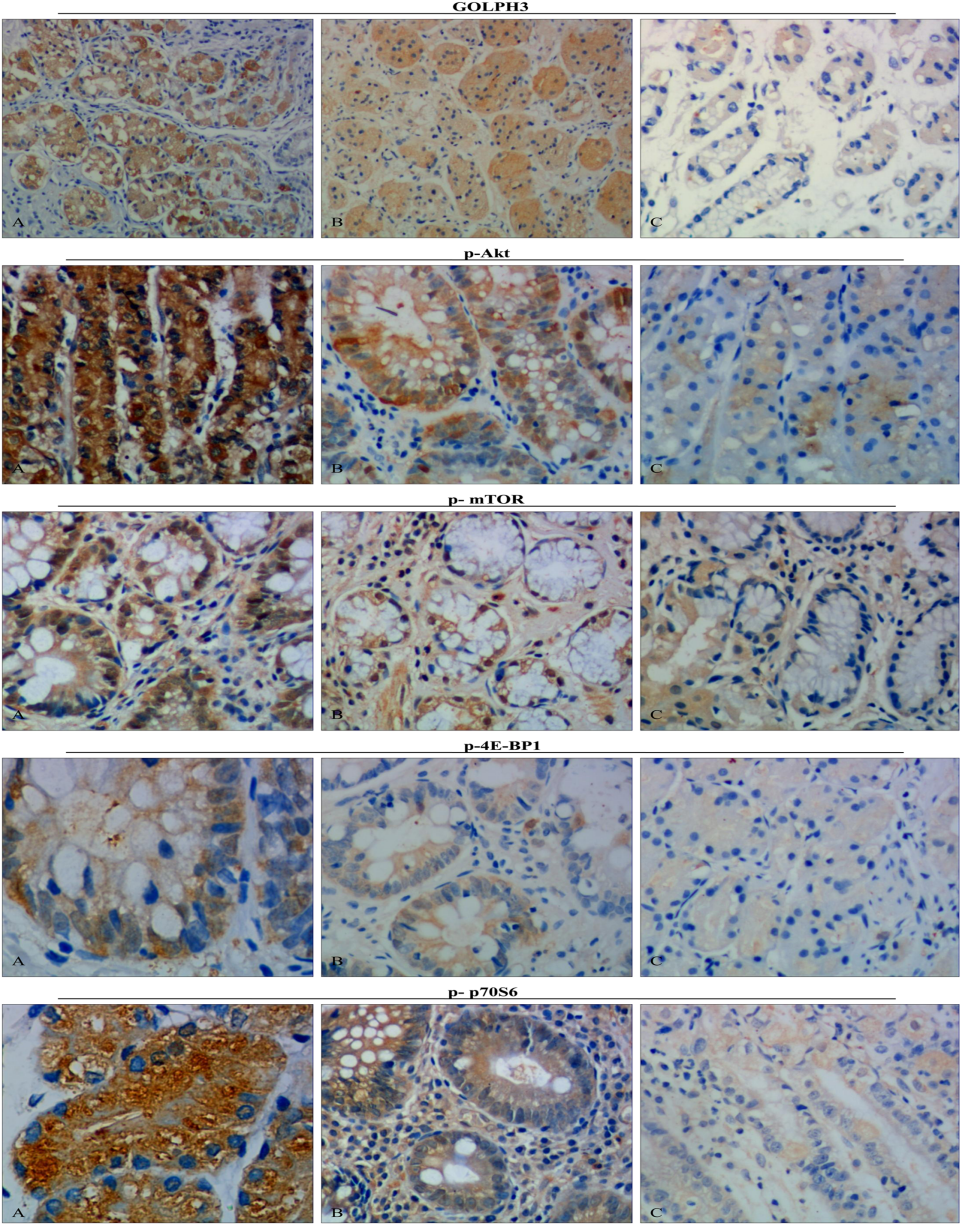
Representative GOLPH3, p-Akt, p-mTOR, p-4E-BP1 and p-p70S6 immunohistochemistry images in different tissues (brown granules, original magnification×400). **A**: The gastric cancer group **B**: The carcinoma-adjacent tissue group **C**: The paired normal tissue group. Representative tissue sections from the gastric cancer group (n = 80), the carcinoma-adjacent tissue group (n = 80) and the paired normal tissue group (n = 80). Immunohistochemistry results demonstrated that the GOLPH3, p-Akt, p-mTOR and p-4E-BP1 were most expressed in the nucleus, the p-p70S6 was most expressed in the cytoplasm. The GOLPH3, phosphorylated Akt-mTOR Signaling pathway, p-4E-BP1 and p-p70S6 were highly expressed in gastric cancer groups.

Immunohistochemical detection showed that GOLPH3, p-Akt, and p-4E-BP1 were most common in the cytoplasm, and phosphorylated m-TOR (p-mTOR) was also present in the nucleus at a low level. However, p-p70S6 staining was observed only in the cytoplasm. GOLPH3 and phosphorylated Akt/mTOR pathway proteins were more highly expressed in the gastric cancer group than in the para-carcinoma tissue and paired normal mucosa groups (Figure 1, Table 5). Furthermore, the relationships between the clinicopathological variables and protein expression were examined and are summarized in Table 1, Table 2 and Table 3. A statistical analysis revealed that the positive expression rate of GOLPH3 in the gastric cancer group strongly correlated with histological grade (*p* = 0.011), depth of invasion (*p* = 0.007), distant metastasis (*p* = 0.002), and lymph node involvement (*p*<0.004), whereas it did not significantly correlate with age or gender (*p* = 0.801 and 0.833, respectively). Interestingly, the high expression levels of p-Akt, p-mTOR, p-4E-BP1 and p-p70S6 were observed to be consistent with high GOLPH3 expression.

**Table 5:** The protein expression levels of GOLPH3, p-AKT1, p-mTOR, p-4E-BP1 and p-p70S6 in different tissues.

	Gastric cancer			para-carcinoma			paired normal gastric			p-value	χ^2^-value
	tissues (n = 80)			tissues (n = 80)			mucosa (n = 80)				
	High	Low	Rate	High	Low	Rate	High	Low	Rate		
**GOLPH3**	60	20	75.00%	35	45	43.75%	10	70	12.50%	0.000*	63.492
**p-AKT1**	54	26	67.50%	36	44	45.00%	20	60	25.00%	0.000*	29.136
**p-mTOR**	65	15	81.25%	31	49	38.75%	12	68	15.00%	0.000*	80.863
**p-4E-BP1**	62	18	77.50%	45	35	56.25%	21	59	26.25%	0.000*	54.691
**p-p70S6**	61	19	76.25%	20	60	37.50%	13	67	16.25%	0.000*	60.305

The GOLPH3, p-AKT1, p-mTOR, p-4E-BP1 and p-p70S6 expression levels were evaluated based on the aggregate scores of the total percentage of positively stained tumor cells and the Immunohistochemistry staining intensity. High: the aggregate scores ≥4; Low: the aggregate scores <4; Rate as the high aggregate scores numbers/the total numbers. Rate of the protein expression levels in different tissues were estimated using the chi-squared test.

*Significant at the level of *p*<0.05.

### mRNA expression of GOLPH3 and mTOR signaling pathway-related genes by RT-PCR

**Figure 2:**
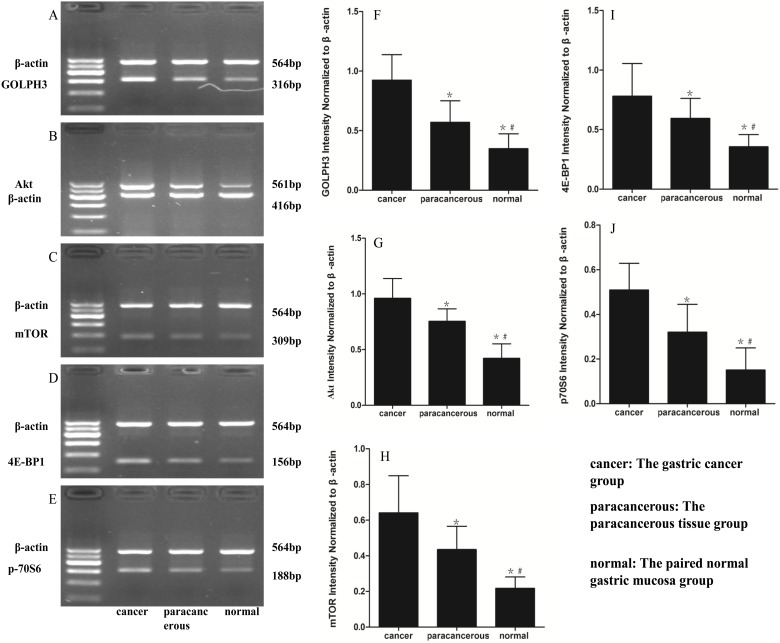
Representative images showed semi-quantitative RT-PCR for GOLPH3, Akt, mTOR, 4E-BP1 and p70S6 transcription. **cancer**: The gastric cancer group (n = 80); **paracancerous**: The paracancerous tissue group (n = 80); **normal**: The paired normal gastric mucosa group (n = 80). **A**, **B**, **C**, **D** and **E**: The mRNA levels of GOPLH3, Akt, mTOR, 4E-BP1, p70S6 in the different measured by RT-PCR, respectively. **F**, **G**, **H**, **I** and **J**: Densitometric quantification of GOLPH3, Akt, mTOR, 4E-BP1 and p70S6 mRNA expression in different tissues, respectively. The mRNA expression was significantly different with each other and peaked at cancer. The data are presented as the mean ± the SE. *p<0.05 versus cancer, ^#^ *p*<0.05 versus paracancerous.

We extracted RNA from gastric cancer, para-carcinoma tissue, and matched normal tissue samples from eighty patients with gastric cancer. The expression levels of GOLPH3 and mTOR signaling pathway-related genes, including Akt, mTOR, eukaryotic translation initiation factor 4E binding protein 1 (4E-BP1), and p70 ribosomal protein S6 kinase (p70S6), were assessed in the three groups by RT-PCR. The expression levels of these genes in gastric carcinoma were higher than in carcinoma-adjacent tissues and significantly higher than in paired normal gastric tissues. The mean density and expression distribution of GOPLH3, mTOR, Akt, 4E-BP1 and p70S6 are shown in Figure 2. A statistical analysis revealed that the expression levels of GOLPH3 and mTOR signaling pathway-related genes were significantly different in different gastric tissues (*p*<0.05).

### Protein expression of GOLPH3 and mTOR signaling pathway related proteins by Western blotting

**Figure 3:**
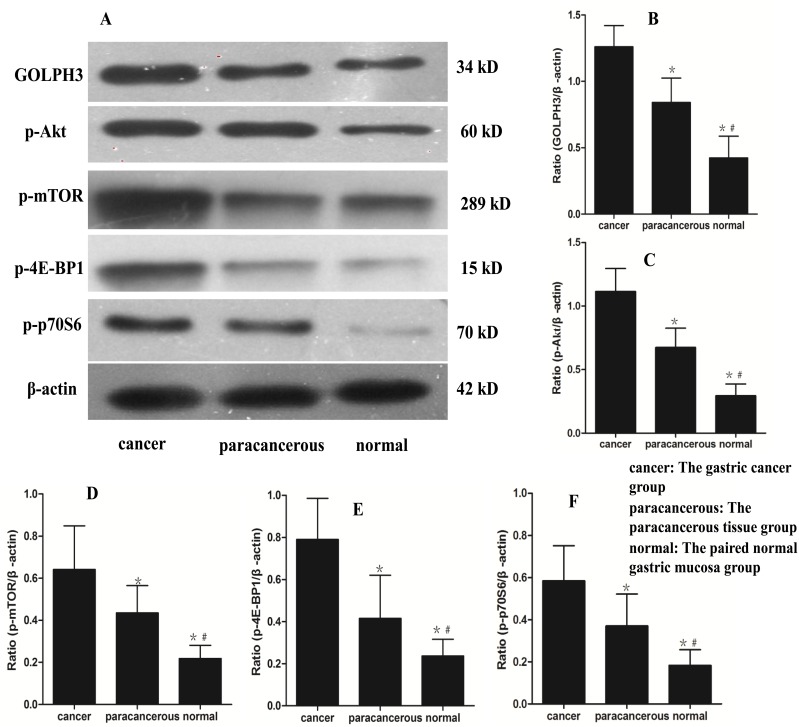
The GOPLH3, p-Akt, p-mTOR, p-4E-BP1 and p-p70S6 protein levels in the different tissues. **A**: The protein levels of GOPLH3, p-Akt, p-mTOR, p-4E-BP1 and p-p70S6 in the different measured by western blotting. **B**, **C**, **D**, **E and** **F**: The GOPLH3, p-Akt, p-mTOR, p-4E-BP1 and p-p70S6 protein levels in the different tissues, respectively. **cancer**: The gastric cancer group (n = 80); **paracancerous**: The paracancerous tissue group (n = 80); **normal**: The paired normal gastric mucosa group (n = 80). The data are presented as the mean ± the SE. *p<0.05 versus cancer, ^#^ *p*<0.05 versus paracancerous.

Western blotting analysis showed that GOLPH3, p-mTOR, p-Akt1, p-4EB-P1 and p-p70S6 were also expressed in the gastric cancer, carcinoma-adjacent, and paired normal gastric mucosa groups. Interestingly, a progressive increase was observed in the mean values of the aforementioned protein expression levels from the paired normal gastric mucosa group to the gastric cancer group (Figure 3), and there was a significant difference between the groups (*p*<0.05).

### Correlations between the expression of GOLPH3 and the signaling signaling pathway-related proteins

Pearson’s correlation analysis revealed a strong correlation between GOLPH3 expression and that of the signaling pathway (Table 6). A statistical analysis showed that the Pearson product moment correlation coefficients were all positive in the gastric cancer group. Interestingly, GOLPH3 expression was strongly associated with p-mTOR, p-4E-BP1 and p-p70S6 (*r* = 0.410, 0.203 and 0.128, respectively, *p*<0.05) expression, and a positive correlation between GOLPH3 and p-Akt was nearly significant (*p* = 0.054). Meanwhile, strong correlations were observed between the expression of p-4E-BP1 and the above-mentioned proteins with the exception of p-mTOR. Phosphorylated mTOR expression was also positively correlated with p-Akt expression (*r* = 0.521, *p* = 0.027). Phosphorylated p70S6 expression was also correlated with the expression of p-Akt (*r* = 0.274, *p*<0.001) and p-mTOR (*r* = 0.451, *p* = 0.007).

**Table 6:** Correlations between the expressions of GOLPH3, p-AKT1, p-mTOR, p-70S6, and p-4E-BP1.

	GOLPH3	p-AKT1	p-mTOR	p-70S6	p-4E-BP1
	(*P*-value)	(*P*-value)	(*P*-value)	(*P*-value)	(*P*-value)
**GOLPH3**					
**p-AKT1**	0.054				
	(*r* = 0.258)				
**p-mTOR**	0.033*	0.027*			
	(*r* = 0.410)	(*r* = 0.521)			
**p-70S6**	0.057	<0.001*	0.007*		
	(*r* = 0.128)	(*r* = 0.274)	(*r* = 0.451)		
**p-4E-BP1**	0.014*	0.037*	0.103	<0.001*	
	(*r* = 0.203)	(*r* = 0.350)	(*r* = 0.187)	(*r* = 0.341)	

Abbreviations: GOLPH3, Golgi phosphoprotein 3; p-Akt1, phosphorylated v-akt murine thymoma viral oncogene homolog 1; p-mTOR, phosphorylated mammalian target of rapamycin; p-p70S6, phosphorylated p70 ribosomal protein S6 kinase; p-4E-BP1, phosphorylated 4E-binding protein-1; *r*, pearson product moment correlation coefficient.

*Significant at the level of *p*<0.05.

## Discussion

Although the incidence of gastric cancer has declined in recent years [2], it remains a serious threat to human health. Most gastric cancer patients are diagnosed in advanced stages and tend to present with tumor invasion and metastasis. Over the past decade, no great improvements have been achieved regarding the early diagnosis and treatment of gastric cancer. An understanding of the molecular biology underlying gastric cancer is still lacking. Since 2009, GOLPH3 has received considerable attention as an oncogene. This protein localizes to the cytoplasmic face of the trans-Golgi and has been closely associated with tumorigenicity [3], [8]. Large studies have found that GOLPH3 overexpression occurs in several human cancers, including epithelial ovarian carcinoma, renal cell carcinoma, glioblastoma multiforme, esophageal squamous cell carcinoma, and oral tongue cancer [12]–[16]. Evidence suggests that GOLPH3 is involved in tumorigenesis and correlates with poor prognosis. Furthermore, one study reported that GOLPH3 overexpression is associated with poor clinical outcome in gastric cancers [17]. However, the precise mechanisms underlying this relationship are unclear, and the exact molecular mechanism by which GOLPH3 is involved in gastric cancer tumorigenesis, invasion and metastasis is poorly understood. To investigate the potential molecular mechanism by which GOLPH3 is involved in gastric cancer, we first used immunohistochemistry to locate GOLPH3 expression in different gastric tissues. We found that GOLPH3 was primarily localized to the cytoplasm, and it was expressed in gastric cancer, para-carcinoma and paired normal gastric tissues. Interestingly, the rates of high GOLPH3 expression were 75.00% (60 out of 80) in gastric cancer tissues, 43.75% (35 out of 80) in carcinoma-adjacent tissues and 12.50% (10 out of 80) in normal tissues, respectively (Table 5). Moreover, GOLPH3 over-expression was observed in gastric cancer tissue and was highly related to gastric cancer invasion and metastasis. Our results suggested that high GOLPH3 expression was significantly related to histological grade (*p* = 0.015), depth of invasion (*p*<0.001), lymph node metastasis (*p*<0.001), and distant metastasis (*p* = 0.006) in gastric cancer tissues (Table 1). In addition, Western blotting and RT-PCR were performed to evaluate GOLPH3 expression at the protein and mRNA levels. Interestingly, the results were similar to those observed in the immunohistochemistry analysis. The above findings indicate that increased GOLPH3 expression is closely associated with the incidence of gastric cancer, and GOLPH3 may be involved in the gastric cancer invasion and metastasis.

Recently, an increasing number of signal transduction pathways have been reported to be involved in tumor invasion and metastasis. Mammalian target of rapamycin (mTOR), which is a serine/threonine kinase downstream of the phosphatidylinositol 3-kinase (PI3K)/Akt signaling pathway and includes mTORC1 (mTOR/RAPTOR) and mTORC2 (mTOR/RICTOR), regulates protein synthesis, cell proliferation, cell metabolism and differentiation [18], [19]. Activated mTORC1 sequentially directly activates downstream targets, including p70S6 kinase (S6K) and eukaryotic initiation factor 4E-binding protein 1 (4E-BP1), and mTORC2 has been shown to play a critical role in Akt phosphorylation at Ser473, resulting in the full activation of Akt [20], [21]. Although Akt directly activates mTOR/RAPTOR, it may also directly or indirectly activate the p70S6 kinase/4EBP1 pathway this pathway by phosphorylating the tumor suppressor TSC2 [22]. The Akt/mTOR pathway has been shown to be frequently activated in various malignant tumors, including breast cancer, urothelial carcinoma, ovarian cancer, pancreatic neuroendocrine tumors, human medulloblastoma, melanoma, endometrial cancer and hepatocellular carcinoma; therefore, this pathway represents a possible therapeutic target in many cancers [23]–[30]. In our study, RT-PCR and Western blotting revealed that the mRNA and protein expression levels of p-Akt, p-mTOR, p-4E-BP1 and p-p70S6 in the gastric cancer group were significantly higher than in the para-carcinoma tissues and paired normal gastric tissue groups (Figure 2, Figure 3). To determine whether the expression levels of p-Akt, p-mTOR, p-4E-BP1 and p-p70S6 were associated with critical clinicopathological characteristics, we performed further analyses. In this study, 81.25% of the 80 patients with gastric cancer had high p-mTOR expression, 77.50% had high p-4E-BP expression, 76.25% had high p-p70S6 expression, and only 75.00% had high p-Akt expression (Table 5). Surprisingly, an immunohistochemical analysis revealed that the expression levels of these protein expressions were significantly associated with the depth of invasion, lymph node and distant metastasis, and histological grade but were not associated with gender or age (Table 1, Table 2 and Table 3). These results suggest that the Akt/mTOR pathway is activated in gastric cancer and may play an important role in gastric cancer tumorigenesis, invasion and metastasis.

Recently, Scott et al. revealed that GOLPH3 can promote cell transformation and tumor growth by constitutively activating the mTOR signaling pathway in melanoma cells and that GOLPH3 stimulates the mTOR signaling pathway via mTORC1 and mTORC2 complexes, thereby promoting tumor cell growth and proliferation [8]. However, it is unclear whether this phenomenon also occurs in gastric cancer tissues. To our knowledge, our study is the first to explore the pathogenesis of GOLPH3 promoted tumorigenesis, invasion and metastasis of gastric cancer. Our data show a significant positive relationship between the expression of GOLPH3 and p-mTOR, p-4E-BP1, and p-p70S6 (*r* = 0.410, 0.203 and 0.128, respectively, *p*<0.05). However, the correlation between the expression of GOLPH3 and p-Akt was not significant (*r* = 0.258, *p*>0.05) (Table 6). In particular, significantly higher GOLPH3 expression was accompanied by high expression of p-mTOR, p-4E-BP1, and p-p70S6 in gastric cancer tissues, and this relationship was significantly associated with the depth of invasion, histological grade, and lymph node and distant metastasis. Our results revealed various relationships between GOLPH3 and Akt/mTOR signaling proteins, and the positive association between GOLPH3 and p-Akt was nearly significant. The specific causes of this relationship between GOLPH3 and p-Akt are unclear and should be further investigated. We speculate that the sample size may have been too small. In addition, GOLPH3 may primarily activate the Akt/mTOR signaling pathway via the activated mTORC1 complex rather than the mTORC2 complex, resulting in p70S6 and 4E-BP1 phosphorylation, thereby affecting gastric cancer. These results indicate that GOLPH3 may be involved in gastric cancer tumorigenesis, invasion and metastasis via abnormal activation of the Akt/mTOR signaling pathway.

In conclusion, this study detected the high expression levels of GOLPH3, p-Akt, p-mTOR, p-4E-BP1 and p-p70S6 in the gastric cancer group strongly which correlated with histological grade, depth of invasion, distant metastasis, and lymph node involvement by clinicopathological variables analysis and immunohistochemistry. Furthermore, the expression of GOPLH3 is highly correlated with the activation of Akt/mTOR signaling pathway in human gastric cancer samples. Based on these results, we speculate that GOLPH3 might be affected progression and metastasis of gastric cancer through the activation of Akt/mTOR signaling pathway. Moreover, we propose that the inhibition of the expression of genes in the GOLPH3 and mTOR signaling pathway may represent a novel target for gastric cancer therapy. Finally, the invasion and migration mechanisms of GOLPH3 in gastric cancer still need further investigation.
